# Efficacy of 1064‐Nm Picosecond Nd: YAG Laser in Treating Facial Photoaging and Improving Skin Barrier Enhanced by a Multi‐Beneficial Composition Formulation: A Blinded Randomized Clinical Trial Study

**DOI:** 10.1111/jocd.70886

**Published:** 2026-05-05

**Authors:** Yixuan He, Manru Ning, Feifei Wang, Yihuai Liang, Shuxian Yan

**Affiliations:** ^1^ Sino United Health Shanghai China; ^2^ Yunnan Characteristic Plant Extraction Laboratory Co., Ltd. Kunming Yunnan China; ^3^ Yunnan Botanee Bio‐Technology Group Co., Ltd. Kunming Yunnan China

**Keywords:** 1064‐nm picosecond laser, photoaging, skin physiology

## Abstract

**Background:**

In recent years, skin photoaging has received increasing attention, and laser therapy has become the most commonly used treatment due to its safety and efficacy; emerging evidence suggests that adjunctive “integrated skincare” may enhance treatment benefits by reducing post‐procedural reactions and supporting skin barrier repair.

**Objective:**

The split‐face, double‐blind, randomized, placebo‐controlled, monocentric clinical trial was conducted to validate the potential efficacy of anti‐photoaging and skin repair of a multifunctional serum formulation following a one‐session therapy of 1064‐nm picosecond Nd:YAG laser.

**Methods:**

A total of twenty‐four participants requesting picosecond laser therapy to address age‐associated facial alterations were recruited for the study. Following a two‐week washout period, participants underwent a single laser session on Day 1 (T1d) and applied either the multi‐beneficial serum or the placebo to the designated facial side twice daily for eight weeks. Their facial skin manifestations were assessed by two dermatologists at baseline, T0, T15d, T29d, and T57d, and the Symptom Score Reduction Index (SSRI) was analyzed later. Non‐invasive measurements and self‐assessments were also administered at each visit.

**Results:**

Twenty‐four women aged 30–48 years completed the trial. The scores for all other manifestations of skin photoaging, with the exception of coarse wrinkles, decreased on both the test and control sides throughout follow‐up. Based on SSRI values for individual photoaging signs, clinical efficacy rates for the test sides were superior to their counterparts. Regarding skin barrier function, decreasing transepidermal water loss and increasing stratum corneum hydration were observed on the test side during the period of recovery, with significant intergroup differences on T29d and T57d. Skin tone indicators (erythema index, melanin index, and tone evenness) rose shortly after the laser treatment and then declined on both sides; the magnitudes of reductions were greater on the test sides than on the control ones. Even though the improvement of skin surface evenness was found due to the therapy, the test sides exhibited more favorable changes than the controls, for instance, a reduction in the area proportion of crow's feet. No adverse events related to the test product were found during the study.

**Conclusion:**

The test serum enhanced the efficacy of 1064‐nm picosecond laser treatment for facial photoaging and supported skin barrier recovery.

**Abbreviations**
EI: erythemal index
HA: hyaluronic acid
MAPK: mitogen‐activated protein kinases
MI: melanin index
NF‐κB: nuclear factor κB
NO: nitric oxide
Nrf2: nuclear factor erythroid 2‐related factor 2
PIH: post‐inflammatory hyperpigmentation
SCH: stratum corneum hydration
SE: standard error
SSRI: Symptom Score Reduction Index
TEWL: transepidermal water loss
TNF‐α: tumor necrosis factor‐α

## 1 Introduction

In recent years, skin photoaging has received increasing attention. It is often accompanied by a series of pathological and physiological changes in the epidermis and dermis, causing various changes in skin texture, deepened wrinkles, and enlarged cutaneous pores. Therapies for photoaging include drugs, lasers, and surgical treatment [1]. Among them, the non‐invasive laser therapy has become the most commonly used treatment due to its low risks and good efficacy. The 1064‐nm picosecond Nd:YAG laser equipped with a microlens array, owing to its ultrashort pulse duration, can produce exceptionally high peak power, which induces laser‐mediated optical breakdown and subsequent dermal remodeling processes, including neo‐collagenesis and neo‐elastinogenesis [2]. Furthermore, the effectiveness of laser delivery systems has been assessed in clinical trials [3]. Nevertheless, the procedure carries a potential risk of post‐inflammatory hyperpigmentation (PIH) [4], especially among individuals with Fitzpatrick skin type IV, which is prevalent in the Chinese population [5].

People used to seek interventions to reduce wrinkles and restore volume to their face through minimally invasive methods; however, today they aim high in the quest for their well‐being and for safer and more durable solutions to prevent skin aging [6]. This shift led to the emergence of the ‘integrated skincare’ concept [7], which involves the use of formulations containing hydrolyzed hyaluronic acid (HA) and a combination of other compounds such as antioxidants, growth factors, and plant extracts to enhance skin quality [8, 9] to synergistically reduce adverse reactions and promote skin barrier repair [10]. Numerous plant‐derived extracts with anti‐aging potential have been identified, each acting on distinct biological pathways involved in skin aging. For instance, extracts such as *paeonia suffruticosa* [11], *cordyceps sinensis* [12], and *ergothioneine* [13] modulate the extracellular matrix; *acmella oleracea extract* [14] promotes muscle relaxation and recovery of contractile function; *kappaphycus alvarezii extract* [15] mitigates telomere shortening and delays dermal fibroblast senescence; while *Morus alba extract* suppresses mitogen‐activated protein kinase (MAPK) activity and demonstrates wound‐healing potential in animal models. All these active ingredients are incorporated into the test multi‐component formula serum, which is expected to accelerate the recovery of skin lesions from microdamage during the inflammatory phase.

This study was designed to comprehensively assess both the immediate and long‐term outcomes of 1064‐nm picosecond laser treatment, administered alone or in combination with a multi‐component anti‐aging formulation. Evaluations included clinical assessments, non‐invasive biophysical analyses, and participant self‐reported questionnaires. The short‐term outcomes were assessed using transepidermal water loss (TEWL) and stratum corneum hydration (SCH), while long‐term effects were evaluated across two aspects, including skin tone and skin surface evenness.

## 2 Patients and Methods

### 2.1 Ethics

The study was approved by the Shanghai Ethics Committee for Clinical Research (ApprovalNo: SECCR/2023–128‐01). The clinical trial was registered at ClinicalTrials.gov (RegistrationNo: NCT06188338). Before any study‐related procedures or measurements, written informed consent was obtained from all participants. Participants were fully informed about the purpose of the study, the procedures involved, potential risks, and their right to withdraw at any time without any consequences.

### 2.2 Study Design

This investigation was designed as a randomized, double‐blind, split‐face, placebo‐controlled clinical trial conducted at a single center. Twenty‐four subjects were recruited and completed a two‐week washout phase using only basic skincare products before receiving the 1064‐nm picosecond laser treatment. All participants completed the protocol. Skin biophysical indices, investigator assessments, and self‐assessments were recorded at T0d, T3d, T5d, T8d, T15d, T29d, and T57d.

### 2.3 Participants

Participants who satisfied all inclusion criteria and did not meet any exclusion criteria were enrolled after providing written informed consent: (1) Healthy women aged between 30 and 50 years; (2) Participants presenting with facial skin roughness, fine wrinkles, and dull complexion associated with photoaging; (3) Subjects without itching, tingling or burning on the face; (4) Subjects with no difference in the level of facial photoaging bilaterally, and the overall facial photoaging score is > 2; (5) Participants who agreed to apply the test products on each side of the face according to the split‐face design for 56 consecutive days. (6) Participants fully understood the purpose and procedures of the study and provided a written informed consent form (ICF). Exclusion criteria: (1) Subjects who have received anti‐aging aesthetic medical treatment within the past 1 year; (2) Female participants who are currently pregnant, nursing, or planning pregnancy during the study period; (3) Participants with anti‐allergy medication history; (4) Participants who had taken part in any other clinical study within the previous month; (5) Any Participants those the investigator considers ineligible.

### 2.4 Treatment Protocol

After enrollment, participants' faces were randomly assigned to a test side and a control side using a random number table, and the outcome evaluators were not involved in the randomization process. All participants were asked to stop using their regular skincare products two weeks prior to treatment. Standardized skincare items, including sunscreen, cleansing foam, and moisturizing emulsion, were supplied for consistent use during the study period.

Before the initiation of the therapeutic intervention, a topical anesthetic (lidocaine cream) was applied to the treatment area for 40 min. Participants then received a single 1064‐nm picosecond laser treatment (Picocare, WONTECH Co. Ltd., Daejeon, Korea) on both sides of the face, performed by a dermatologist on day T1d. The treatment was delivered using a microlens array (MLA) handpiece. Each patient underwent three passes at a fluence of 1.0 J/cm^2^ with a repetition rate of 10 Hz and a pulse duration of 450 ps, and each split‐face treatment involved 3 000 laser spots. The desired clinical endpoint was the appearance of erythema and acicular purpura.

Immediately after laser treatment, participants applied the anti‐aging formulation to the designated test side and the placebo formulation to the control side, maintaining this regimen twice daily for 56 consecutive days. The facial midline—defined by anatomical reference points including the glabella, nasal bridge, philtrum, and chin—was used to demarcate the two treatment areas. In the split‐face controlled trial, to prevent cross‐use of the products, the external packaging is labeled with the Chinese characters (literally, “left” or “right”) to strictly designate the application areas for the respective facial halves. Furthermore, the product assigned to the left facial half is to be handled exclusively with the left hand, and the product for the right facial half is to be handled exclusively with the right hand.

### 2.5 Formulation

The test item is a commercially available anti‐aging serum containing a proprietary complex of *cordyceps sinensis* extract, *aeonia suffruticosa* extract, *kappaphycus alvarezii* extract, ergothioneine, *acmella oleracea* extract, *morus alba* extract, β‐alanyl hydroxyprolyldiaminobutyroyl benzylamide, and palmitoyl hexapeptide‐12, produced by Yunnan Botanee Co. Ltd. In China. To ensure the integrity of the blind design, the placebo used in this trial is identical to the test product in terms of physicochemical properties and sensory characteristics. Specifically, the two are indistinguishable from each other in appearance, odor, packaging materials, labeling information (displaying only the randomization code), and route of administration. The placebo is formulated using the same inactive ingredients as the test product, excluding the actives.

### 2.6 Clinical Evaluation (Primary Endpoints)

The clinical signs of photoaging were scored using a five‐point ordinal scale (0–4), where higher scores indicated worse cases. Six items included tactile roughness, fine lines, coarse wrinkles, mottled pigmentation, and a global score for photoaging adopted from the scale of skin photodamage manifestations, [16] as well as dull and yellowish tone. Two well‐trained dermatologists independent from the study recorded these scores for each side of the face at T0d (before the laser treatment), T15d, T29d, and T57d. Changes in clinical scores were considered the primary endpoint of this study.

The Symptom Score Reduction Index (SSRI) based on scores of clinical evaluation was employed to determine whether the treatment provided a meaningful clinical benefit to one facial half of each participant:

$$\begin{array}{cc} \text{SSRI}=\; & \left(\text{pre‐treatment score}-\text{post‐treatment score}\right)/\\ & \text{pre‐treatment score} \end{array}$$

Clinical efficacy of each photoaging sign was graded as “significantly effective” (SSRI > 0.6), “effective” (0.2 < SSRI ≤ 0.6), “minimally effective” (0 < SSRI ≤ 0.2), and “ineffective” (SSRI = 0). The efficacy rate for each sign was calculated as the percentage of the study participants who achieved a predefined response, i.e., “significantly effective” or “effective”, and then compared between the test and control sides.

It should be noted that the cutoff values of SSRI used to define clinical efficacy were adopted as an exploratory attempt to provide a more intuitive presentation of efficacy differences, though not formally validated yet.

### 2.7 Instrumental Evaluation (Secondary Endpoints)

All evaluations were conducted in a controlled environment (temperature: 21°C ± 1°C; relative humidity: 50% ± 10%). Participants were allowed a 30‐min acclimatization period prior to measurement.

At each visit, transepidermal water loss (TEWL) was determined using Tewameter (Courage&Khazaka, Germany). The stratum corneum hydration (SCH) was determined by Corneometer (Courage&Khazaka, Germany). Skin color was recorded using Mexameter MX18 (Courage&Khazaka, Germany), measuring components such as melanin content and erythema level. Facial photographs were obtained using the VISIA imaging system (Canfield Scientific, USA), and then skin tone evenness and facial pore area percentage were quantified using Image‐Pro Plus image analysis software (Media Cybernetics, USA).

Skin wrinkles were analyzed with the PRIMOS system (Canfield Scientific, USA) to quantify both wrinkle volume and surface area percentage of the crow's feet region at T0d, T29d, and T57d.

### 2.8 Sample Size

To increase the statistical power for detecting the intergroup differences of secondary endpoints, an independent sample method instead of the original paired samples design was used to estimate the sample size, aiming for a larger sample size.

Using G*Power software (version 3.1.9.2), with a two‐sided Mann–Whitney test, α = 0.05 and 1 − β = 0.95, based on the expected change in the scores of *Skin Manifestations of Photodamage* in a pre‐test, the minimum required sample size was 20 participants per group. Considering the split‐face, self‐controlled design and a potential 20% dropout rate, a total of 24 participants were planned for inclusion.

### 2.9 Statistical Analysis

Statistical analysis was performed by using SPSS version 22.0. The normality of the data set was assessed using the Shapiro–Wilk test. The intragroup comparison was performed between the baselines and the other visits, using repeated‐measures ANOVA for data normally distributed; otherwise, the Friedman test would be used. Intergroup comparisons based on differences from baselines were performed between the test and control sides, using a paired *t*‐test for data with a normal distribution or the Wilcoxon signed‐rank test for the non‐normally distributed data. Additionally, the Wilcoxon signed‐rank test and the Friedman test were applied to the categorical variables for comparing the intergroup differences and the intragroup differences, respectively. A two‐sided *p*‐value less than 0.05 was considered statistically significant.

To estimate the clinical benefits brought by the test serum, effect sizes with 95% confidence intervals (CIs) were calculated and reported for the endpoints with statistically significant intergroup differences at the final visit.

## 3 Result

### 3.1 Demographic Characteristics of Subjects

Twenty‐four female participants were enrolled, and all completed the study. The mean age was 38.46 ± 5.17 years (range: 30–48 years).

### 3.2 Clinical Evaluation of Skin Photoaging

Clinical scores of skin photoaging are summarized in Table 1. The scores for all other manifestations of skin photoaging, with the exception of “Coarse wrinkles”, decreased with time on both the test and control sides, showing statistically significant differences compared to their baselines (*p* < 0.05). At T57d, although intergroup differences did not reach statistical significance, sum scores on the test sides were consistently lower than those on the control sides across photoaging signs, suggesting an overall better skin status on the test sides.

**TABLE 1:** Blinded investigator–assessed clinical scores for photoaging signs at different time points [sum (minimum, median, maximum)].

Assigned interventions	Skin manifestations	Scores on each visit			
		BL	T15d	T29d	T57d
Test serum (Test sides)	Tactile roughness	69 (2,3,4)	59 (2,2,4)*	56 (1,2,4)***	53 (1,2,4)***
	Coarse wrinkles	32 (0,1,3)	31 (0,1,3)	30 (0,1,3)	29 (0,1,3)
	Fine wrinkles	66 (2,3,3)	57 (1,2,3)**	55 (1,2,3)***	54 (1,2,3)***
	Dull and yellowish tone	55 (1,2,4)	47 (1,2,3)**	45 (1,2,3)**	45 (1,2,3)**
	Mottled pigmentation	55 (1,2,4)	48 (1,2,3)**	45 (1,2,3)**	44 (1,2,3)***
	Overall assessment	74 (3,3,4)	66 (2,3,3)**	61 (2,3,3)***	59 (2,2,3)***
Placebo (Control sides)	Tactile roughness	69 (2,3,4)	62 (2,3,4)**	61 (1,3,4)**	60 (1,2.5,4)**
	Coarse wrinkles	32 (0,1,3)	32 (0,1,3)	32 (0,1,3)	32 (0,1,3)
	Fine wrinkles	65 (2,3,3)	59 (2,2,3)*	58 (2,2,3)**	58 (2,2,3)**
	Dull and yellowish tone	55 (1,2,4)	49 (1,2,3)*	47 (1,2,3)**	47 (1,2,3)**
	Mottled pigmentation	56 (1,2,4)	50 (1,2,4)*	49 (1,2,4)**	49 (1,2,4)**
	Overall assessment	74 (3,3,4)	67 (2,3,3)**	65 (2,3,3)**	65 (2,3,3)**

*Note:* Indicates a statistically significant difference from the baseline. Lower scores indicate better skin condition.

* *p* < 0.05.

** *p* < 0.01.

*** *p* < 0.001.

**FIGURE 1:**
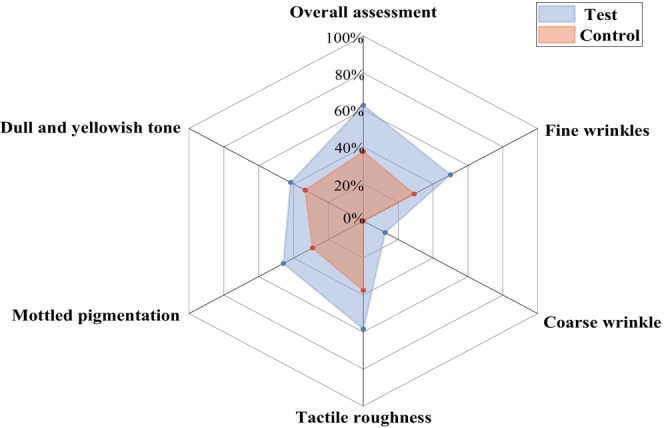
The efficacy rate for each sign of photoaging on the test and control sides at the end of the study.

At the end of the study, the efficacy rates for all symptoms were found to be higher on the test sides than on the control sides (Figure 1). Notably, the efficacy rate differences between the test serum and placebo were 25.0% (95% CI: 4.2%, 45.8%) for “Overall assessment” and 20.83% (0.90% to 40.76%) for “Fine wrinkles”.

### 3.3 Improvement in Skin Barrier

**FIGURE 2:**
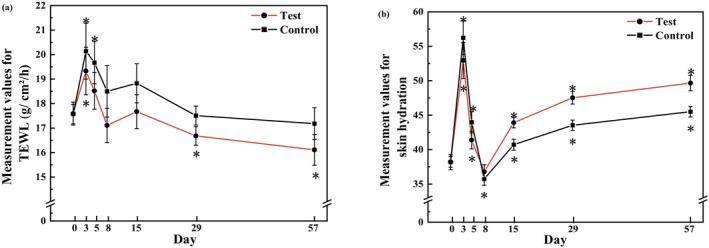
Evaluation of skin barrier–related parameters after 56 days of applying the test and control formulations. (a) Measurement values for TEWL (mean ± SE). (b) Measurement values for SCH (mean ± SE). *p < 0.05, compared with the baseline. SE, standard error.

**FIGURE 3:**
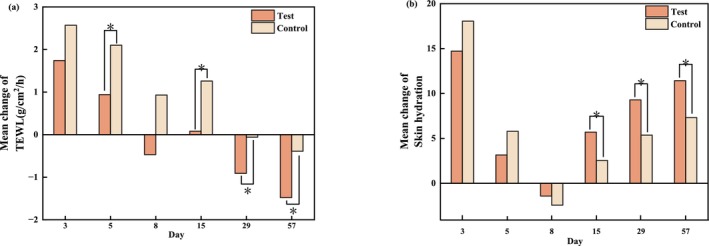
Comparison of skin barrier–related parameters between the treated and control sides at each time point relative to baseline. (a) Mean change in TEWL. (b) Mean change in SCH. Paired *t* tests, *p < 0.05.

TEWL and SCH are commonly used to evaluate the integrity of the skin barrier. Their measurements across the clinical trial and an intragroup comparison at each visit relative to the baselines are shown in Figure 2 and Figure 3 respectively. On the test side, the TEWL values gradually decreased to a lower level than the baseline on T8d and further decreased by 8.42% at the end of the study (*p* = 0.003). However, on the control side, the TEWL values were restored to the baseline level on T29d and showed only a 2.19% decrease from baseline at T57d (*p* = 0.189). When comparing the test side and the control side at the same post‐treatment time points based on differences from baseline, TEWL values on the test sides were significantly lower than those on the control sides at T5d (*p* = 0.022), T29d (*p* = 0.005), and T57d (*p* = 0.003) during the recovery phase. At the end of the study, the mean difference in TEWL between the test sides and the corresponding control ones was −1.10 (−1.67, −0.52) g/cm^2^/h.

Because of postoperative edema, SCH values were significantly increased on both sides at T3d compared with baseline (both *p* < 0.001), indicating a transiently compromised skin barrier, and returned to baseline levels by T8d. Thereafter, SCH gradually increased on both sides relative to baseline. On the test sides, SCH values were significantly higher than baseline at T15d, T29d, and T57d (all *p* < 0.001), whereas on the control sides, significant increases were observed at T15d (*p* = 0.040) and at T29d and T57d (both *p* < 0.001). When comparing the test sides and the control sides at the same post‐treatment time points based on differences from baseline, SCH values on the test sides from T15d to T57d were consistently higher than those on the control sides, suggesting that the test formulation contributed to a more rapid recovery of the skin barrier and improvement of its integrity. At the end of the study, SCH on the test sides increased by 4.12 (2.71, 5.53) compared to their counterparts.

### 3.4 Improvement in Skin Tone

**FIGURE 4:**
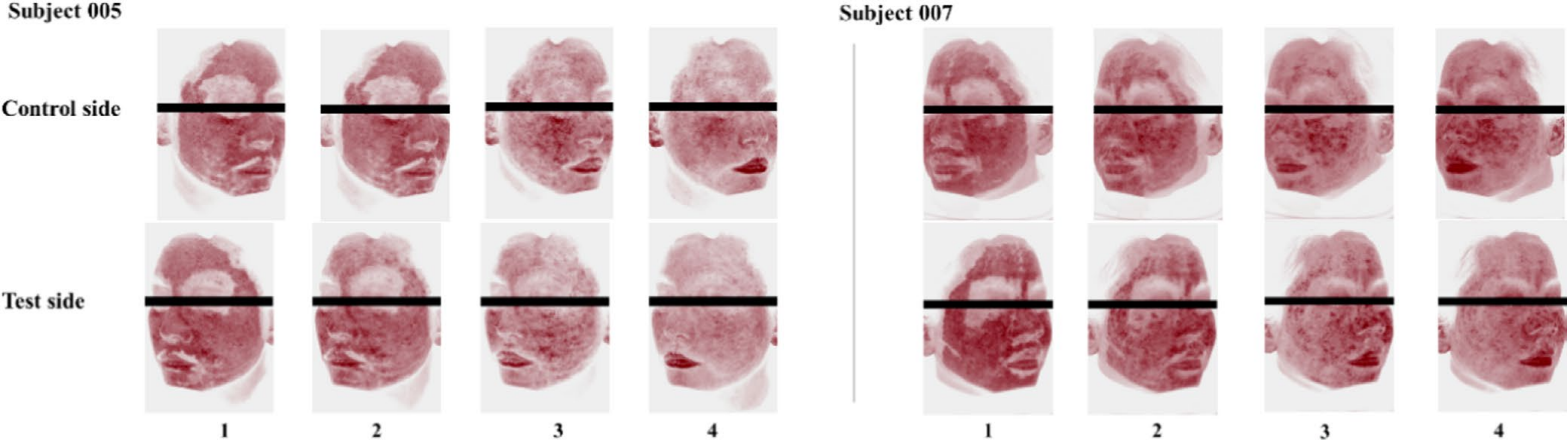
VISIA images of skin condition (red areas) on both the treated and control sides of subject 005 and subject 007. (1) Immediately after laser treatment; (2) Immediately after using the test or control formulations; (3) Three days following laser treatment; (4) Five days following laser treatment.

**FIGURE 5:**
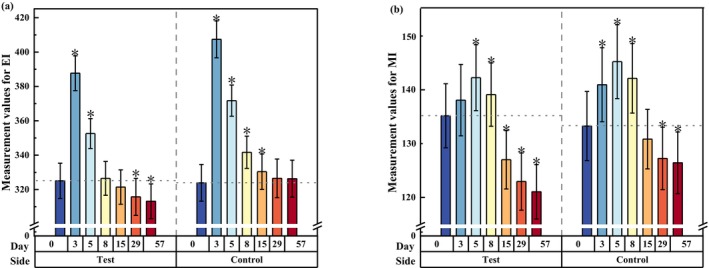
Evolution of skin tone parameters assessed by biophysical analysis after 56 days of treatment with the test and control formulations. (a) Measurement values for EI (mean ± SE). (b) Measurement values for MI (mean ± SE). *p < 0.05, compared with the baseline.

Due to acute stimulation by laser therapy, the skin erythema intensifies, manifested by an increase in the erythema index (EI). The representative examples of red areas on the skin are presented in Figure 4. In the present study, significant elevations in EI values at T3d on both sides were observed compared to their corresponding baselines, i.e., an increase of 19.27% on the test sides (*p* < 0.001) and 25.78% on the control sides (*p* < 0.001) (Figure 5a). Subsequently, a decline in the EI occurred on both sides over time, but the magnitude of reduction was not uniform: The values on the test sides returned to baseline levels by T15d and below the baseline at T57d, whereas those on the control sides remained above baseline levels even at T57d. At the end of the study, based on the differences from baseline, the reduction in EI was found to be greater on the test sides than on the control sides [*p* < 0.001, effect size: −14.32 (−19.46, −9.18)].

The changes in melanin index (MI) values showed a similar temporal pattern (Figure 5b). Relative to baseline, the test sides exhibited a minimal increase of 2.15% at T3d, whereas the control side showed a statistically significantly greater increase of 5.76% (*p* = 0.007). During the recovery phase, MI values on both sides began to drop from T5d onward. At the final assessment, the mean MI decreased by 10.45% from baseline on the test sides (*p* < 0.001) and by 5.13% on the control sides (*p* < 0.001); the magnitude of reduction in MI on the test side was greater than that on the control sides [*p* < 0.001, effect size: −7.28 (−9.06, −5.50)].

**FIGURE 6:**
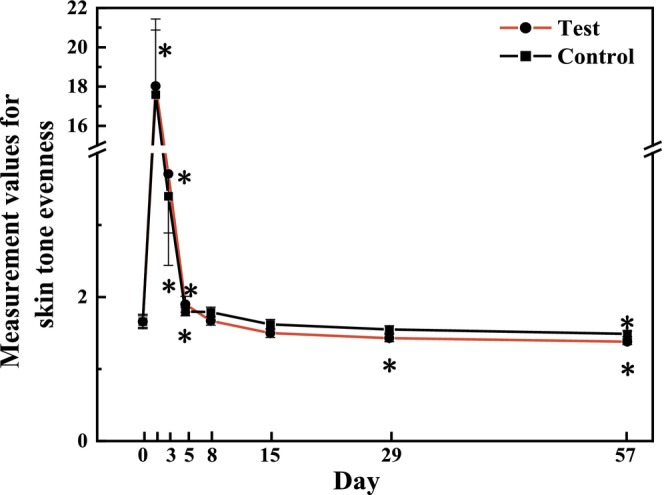
Measurement values for skin tone evenness after 56 days of using test or control formulations (mean ± SE). *p < 0.05, compared with the baseline.

**FIGURE 7:**
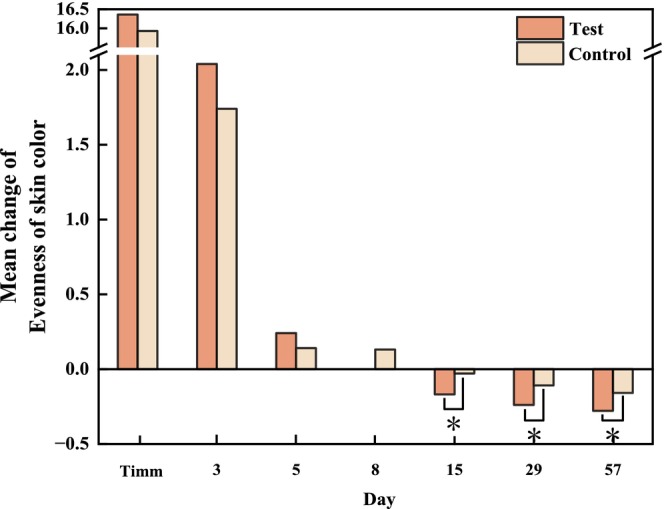
Bilateral comparison of skin tone evenness at each visit relative to the baseline. Paired *t* tests, *p < 0.05.

In VISIA image analysis, evenness refers to the uniformity of facial skin coloration. A lower numerical value represents less difference, meaning a more even skin tone. In the present study, skin tone evenness peaked immediately after the laser therapy, declined during recovery, returning to baseline levels by T15d, and fell below the baseline at T57d (Figure 6). When comparing the test sides and the control sides based on changes from baseline (Figure 7), statistically significant differences were observed at T15d (*p* = 0.025), T29d (*p* = 0.029), and persisted until the final follow‐up [*p* = 0.002, effect size: −0.12 (−0.20, −0.04)], suggesting that the test sides maintained a more favorable skin tone condition throughout the study.

### 3.5 Improvement in Skin Surface Evenness

#### 3.5.1 Measurement of Skin Wrinkles

**FIGURE 8:**
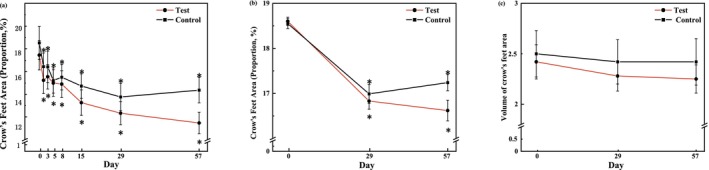
Changes in characteristics of skin wrinkles (crow's feet) for 56 days of treatment with the test and control formulations (a) the percentages of crow's feet area measured by the VISIA system (mean ± SE); (b) the percentages of crow's feet from Primos (mean ± SE); (c) the volumes of crow's feet from Primos (mean ± SE). *p < 0.05, compared with the baseline.

**FIGURE 9:**
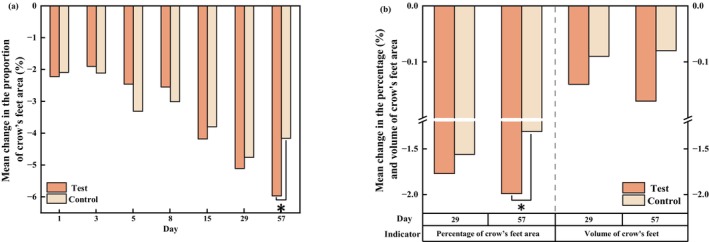
Bilateral comparison of skin wrinkles at the visit relative to the baseline. (a) Mean change in the proportion of crow's feet area. (b) Mean changes in the Primos‐related data. Paired *t* tests, *p < 0.05.

**FIGURE 10:**
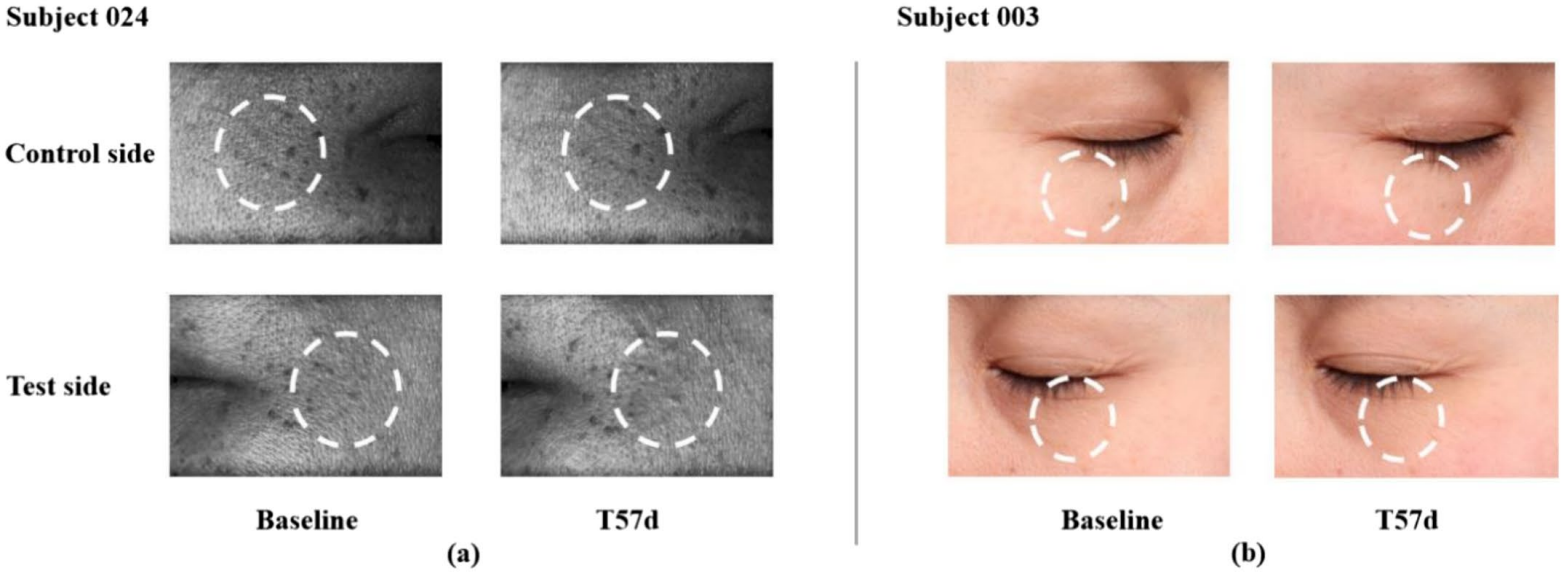
Representative baseline and post‐treatment (T57d) images of wrinkles from two subjects. (a) PRIMOS 3D skin‐surface images of crow's‐feet, acquired using a digital stripe‐projection light source, with a face‐device distance (13 cm) and 90° lateral imaging angles. (b) VISIA images of under‐eye wrinkles, taken under standard white‐light illumination with a fixed face–camera distance (25 cm) and ±45° oblique angles. All images were captured under the consistent settings of lighting, distance, and angle at each visit.

The area proportion of the crow's feet on the test side showed a steady decrease from T3d, and this decline trend was sustained through T57d, with a decrease of 34.23% from 17.44% at baseline to 11.47% on T57d. However, a relatively slight decrease of 22.46% occurred on the control side, from 18.52% to 14.36% (Figure 8a). Additionally, a similar pattern was found in Primos over time. The area percentages and volume of the crow's feet produced by Primos were depicted in (Figure 8b,C). Both of these showed a decreasing trend over 56 days. When comparing the test side and the control side at T57d based on changes from baseline, a statistically significant intergroup difference was observed for the percentage of crow's feet area [*p* = 0.015 for VISIA image analysis, effect size: −1.81% (−3.24%, −0.38%); and *p* = 0.003 for Primos analysis, effect size: −0.67% (−1.07%, −0.28%)], whereas no significant intergroup difference was detected for wrinkle volume (Figure 9). The representative examples of skin wrinkles are presented in Figure 10.

#### 3.5.2 Evaluation of Sebaceous Pores

**FIGURE 11:**
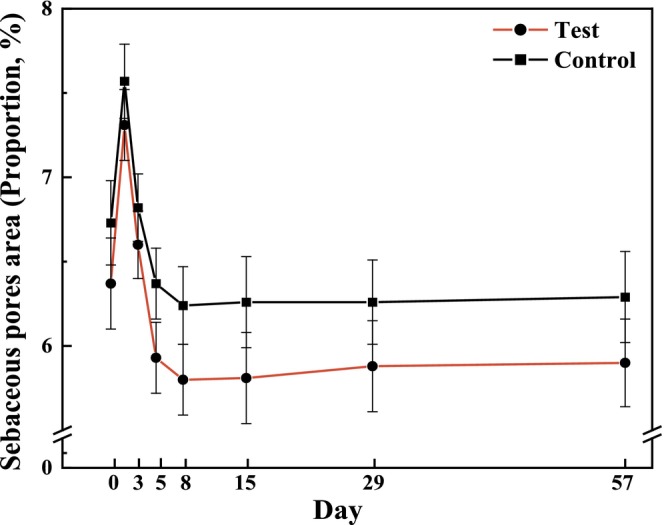
Measurement values for the proportion of sebaceous pores after 56 days of using test or control formulations (mean ± SE).

**FIGURE 12:**
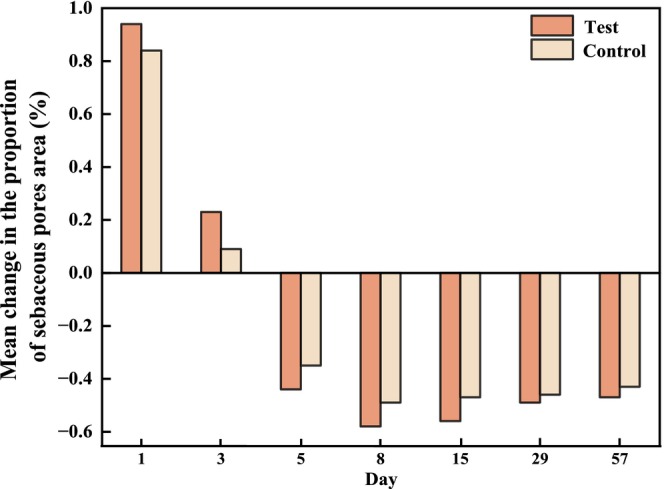
Bilateral comparison of sebaceous pores at the visit relative to the baseline.

The changes in sebaceous pores, characterized by pore area percentage, can be another indicator of skin surface evenness. The percentage of sebaceous pore area on both sides decreased from T5d onward (Figure 11). However, no statistically significant difference in reduction between both sides was observed (Figure 12).

### 3.6 Tolerability

No severe adverse events were reported throughout the study period. Transient erythema, edema, and dryness were observed after treatment; however, these symptoms were mild and resolved spontaneously within 7 days. In addition, these symptoms were attributed to the laser procedure, and no cosmetic product–related adverse reactions were reported. Thus, the test serum did not exhibit a potential for irritation and sensitization.

## 4 Discussion

The 1064‐nm picosecond laser, using thermal energy to disrupt melanin particles, is a widely used technique for pigmented lesions treatment. Beyond its impact on pigmentation, the 1064‐nm picosecond laser also stimulates fibroblast proliferation and collagen production, gradually improving photoaging symptoms, including pigmentation, skin tone, and fine lines. However, there are associated risks, including post‐inflammatory hyperpigmentation and abnormal activation of melanocytes, particularly among individuals of Fitzpatrick skin type IV; prolonged erythema lasting more than three days tends to increase the risk of PIH [5]. Studies have shown that *salvia miltiorrhiza* [17], *morus alba* [18], and *paeonia suffruticosa* [19] in the serum inhibit tyrosinase‐dependent melanogenesis.

Some adverse events, such as erythema, edema, and skin dryness, may occur following picosecond laser, although these symptoms are generally self‐limiting and still negatively impact the quality of life. Therefore, it is necessary to implement appropriate post‐procedural care to reduce downtime. The endogenous antioxidant response pathway plays a crucial role in repairing the epidermal structure and restoring the skin barrier. The active ingredients in the test serum, moutan cortex and ergothioneine, can activate nuclear factor erythroid 2‐related factor 2 (Nrf2) in keratinocytes, thereby enhancing the production of endogenous antioxidants. Additionally, cordyceps extract and moutan cortex suppress the nuclear factor κB (NF‐κB) pathway, inhibit the production of downstream inflammatory mediators, and directly downregulate several pro‐inflammatory cytokines, such as interleukin (IL)‐1β, IL‐6, tumor necrosis factor‐α (TNF‐α), and nitric oxide (NO), thereby achieving anti‐inflammatory effects. Alleviating inflammation is also an effective strategy to prevent PIH.

In addition to its biological effects, picosecond laser treatment may facilitate the transdermal delivery of topical formulations. The ultrashort pulse duration generates a predominantly photomechanical effect, leading to laser‐induced optical breakdown (LIOB) and the formation of transient microscopic channels within the epidermis. These reversible structural alterations temporarily increase skin permeability, thereby enhancing the penetration and retention of topically applied active ingredients without causing significant thermal damage.

Moreover, using the test serum after picosecond laser treatment addresses the challenge of the penetrated epidermal barrier, enhancing the effective delivery of these active ingredients. In this study, the symptoms of facial skin barrier damage observed in patients post‐treatment showed earlier improvement with the multi‐beneficial formula serum, suggesting that the active ingredients in the test product may have played a positive role. Furthermore, the active ingredients in the test serum, palmitoyl hexapeptide‐12 and *morus alba extract*, have been shown to stimulate collagen production, while β‐alanyl hydroxyprolyl‐diaminobutyroyl benzylamide has been confirmed to promote the proliferation of fibroblasts and keratinocytes. These components can synergistically enhance the effectiveness of picosecond laser treatment in addressing fine lines and the loss of elasticity caused by photoaging. In this study, the crow's feet on the test side showed significant improvement compared to the control side at T57d, suggesting that long‐term use of this serum may promote tissue regeneration and restore skin rejuvenation.

Beyond statistical significance, the magnitudes of these changes in the primary and secondary endpoints were considered in the present study. Compared to the controls, the test sides exhibited a visually appreciable change in skin tone evenness (effect size: −0.12) from VISIA image at the final visit, consistent with the reductions in MI (−7.28) and EI (−14.32); likewise, higher efficacy rates of mitigating photoaging signs (overall assessment: +25.0%; fine wrinkles: +20.83%) were corroborated by improvement in the hydration status of skin barrier (TEWL: −1.10 g/cm^2^/h; SCH: +4.12) and in skin surface evenness (decreased proportions of crow's feet area: −1.81% for VISIA image and −0.67% for Primos analysis). Thus, these combined effects underscored the practical benefits of the test serum for overall skin aesthetic quality that can be perceived and valued by individuals.

The main limitations of this study are the small sample size and the relatively short duration of follow‐up. A larger number of participants would improve the robustness of the findings and enhance the statistical power of the analysis. Moreover, a longer observation period (e.g., 12 weeks or half a year) would allow investigating the long‐term benefits of the test serum, particularly in pigment lightening and wrinkle reduction.

## 5 Conclusion

After one session of therapy with a 1064‐nm picosecond laser, continuous use of the multi‐beneficial formula serum can help maintain skin barrier homeostasis and enhance the remedy of photoaging‐induced damage. No adverse events related to the test formulation were observed during the trial. More evidence from rigorously designed clinical trials is warranted to validate these findings.

## Author Contributions

Yixuan He: Conceptualization; Investigation; Writing – review and editing. Manru Ning: Conceptualization; Writing – original draft; Supervision. Feifei Wang: Funding acquisition; Writing – review and editing; Project administration. Yihuai Liang: Conceptualization; Methodology; Writing – review and editing. Shuxian Yan: Conceptualization; Methodology; Investigation; Project administration; Writing – review and editing.

## Funding

This study was supported by the independent research fund of Yunnan Characteristic Plant Extraction Laboratory (2022YKZY006 and 2025YKZY003). We are thankful for the financial support, which was used for the study design, data collection, and data analysis.

## Ethics Statement

The study was approved by the Shanghai Ethics Committee for Clinical Research (ApprovalNo: SECCR/2023–128‐01). The clinical trial was registered at ClinicalTrials.gov (RegistrationNo: NCT06188338). Before any study‐related procedures or measurements, written informed consent was obtained from all participants. Participants were fully informed about the purpose of the study, the procedures involved, potential risks, and their right to withdraw at any time without any consequences.

## Conflicts of Interest

The authors declare no conflicts of interest.

## Data Availability

The data that support the findings of this study are available on request from the corresponding author. Study protocol and all of the individual participant data collected during the trial, after deidentification can be shared, immediately following publication.
